# Genome-Wide Transcriptional Start Site Mapping and sRNA Identification in the Pathogen *Leptospira interrogans*

**DOI:** 10.3389/fcimb.2017.00010

**Published:** 2017-01-19

**Authors:** Anna Zhukova, Luis Guilherme Fernandes, Perrine Hugon, Christopher J. Pappas, Odile Sismeiro, Jean-Yves Coppée, Christophe Becavin, Christophe Malabat, Azad Eshghi, Jun-Jie Zhang, Frank X. Yang, Mathieu Picardeau

**Affiliations:** ^1^Bioinformatics and Biostatistics Hub, Institut Pasteur, C3BIParis, France; ^2^Biology of Spirochetes Unit, Institut PasteurParis, France; ^3^Mutualized Microbiology Platform, Institut Pasteur, Pasteur International Bioresources NetworkParis, France; ^4^Department of Biology, Manhattanville CollegePurchase, NY, USA; ^5^CITECH, Institut Pasteur, Plate-forme Transcriptome et Epigenome, Pole Biomics – CITECHParis, France; ^6^Department of Microbiology and Immunology, Indiana University School of MedicineIndianapolis, IN, USA

**Keywords:** leptospirosis, spirochetes, promoter, transcription factors, RNA

## Abstract

*Leptospira* are emerging zoonotic pathogens transmitted from animals to humans typically through contaminated environmental sources of water and soil. Regulatory pathways of pathogenic *Leptospira* spp. underlying the adaptive response to different hosts and environmental conditions remains elusive. In this study, we provide the first global Transcriptional Start Site (TSS) map of a *Leptospira* species. RNA was obtained from the pathogen *Leptospira interrogans* grown at 30°C (optimal *in vitro* temperature) and 37°C (host temperature) and selectively enriched for 5′ ends of native transcripts. A total of 2865 and 2866 primary TSS (pTSS) were predicted in the genome of *L. interrogans* at 30 and 37°C, respectively. The majority of the pTSSs were located between 0 and 10 nucleotides from the translational start site, suggesting that leaderless transcripts are a common feature of the leptospiral translational landscape. Comparative differential RNA-sequencing (dRNA-seq) analysis revealed conservation of most pTSS at 30 and 37°C. Promoter prediction algorithms allow the identification of the binding sites of the alternative sigma factor sigma 54. However, other motifs were not identified indicating that *Leptospira* consensus promoter sequences are inherently different from the *Escherichia coli* model. RNA sequencing also identified 277 and 226 putative small regulatory RNAs (sRNAs) at 30 and 37°C, respectively, including eight validated sRNAs by Northern blots. These results provide the first global view of TSS and the repertoire of sRNAs in *L. interrogans*. These data will establish a foundation for future experimental work on gene regulation under various environmental conditions including those in the host.

## Introduction

Pathogenic *Leptospira* spp. are the etiologic agents of leptospirosis, a disease manifesting as a wide range of clinical symptoms. A recent study estimates that more than one million severe cases of leptospirosis occur annually, including 60,000 deaths (Costa et al., 2015). Rats are asymptomatic reservoirs of pathogenic *Leptospira* spp. and contribute to the transmission cycle of the bacteria via bacterial shedding through the urinary tract to environmental sources. Other mammalian species, wild, and domestic, can also serve as reservoirs and present a range of mild to fatal disease manifestations. *Leptospira* are typically transmitted to humans by exposure to environmental surface water that is contaminated with the urine of infected animals. Leptospirosis has emerged as a major public health problem, especially in the developing world, due to global climate changes and urban sprawl.

Our current understanding of the virulence mechanisms and more generally the biology of pathogenic *Leptospira* remains largely unknown, partly due to the lack of efficient genetic tools and fastidious *in vitro* culturing of pathogenic *Leptospira* spp. (Ko et al., 2009). The transmission cycle of *Leptospira* exposes the bacteria to drastically different environments and *Leptospira* must be able to adapt to such disparities to retain viability. Adaptive responses of *Leptospira interrogans* have been analyzed by whole-genome microarrays to determine global changes in transcript levels of *L. interrogans* in response to interaction with phagocytic cells (Xue et al., 2010), temperature (Lo et al., 2006; Qin et al., 2006), osmolarity (Matsunaga et al., 2007), iron depletion (Lo et al., 2010), and serum exposure (Patarakul et al., 2010), which are relevant to changes that occur during infection. These transcriptome studies have shown that *Leptospira* spp. are capable of responding to a diverse array of environmental signals. However, the molecular mechanisms of bacterial adaptation and regulatory networks remain unknown.

In a recent study, high-throughput RNA sequencing of *L. interrogans* serovar Copenhageni cultivated within dialysis membrane chambers (DMCs) implanted into the peritoneal cavities of rats allowed the identification of 11 putative small non-coding RNAs (sRNAs) whose functions remain to be determined (Caimano et al., 2014). Other potential regulatory non-coding RNAs identified in *Leptospira* spp. include an RNA thermometer (Matsunaga et al., 2013) and riboswitches (Ricaldi et al., 2012; Fouts et al., 2016; Iraola et al., 2016). In addition to transcription factors, *Leptospira* species have several alternative sigma factors that are known to be important for environmental adaptation and bacterial virulence in other bacteria (Kazmierczak et al., 2005), such as σ^54^ (σ^N^, RpoN) involved in nitrogen utilization and many cellular and environmental responses, σ^28^ (σ^F^, FliA) involved in flagella gene expression, and several extracytoplasmic function (ECF) sigma factors σ^24^ (σ^E^) involved in regulation of membrane and periplasmic stress.

To improve genome annotation and promote our understanding of *L. interrogans* gene structures and RNA-based regulation, we present here a transcriptional map of the *L. interrogans* genome including the characterization of primary transcription start sites (TSS), alternative TSS, operon organization, and specific DNA sequence motifs located in promoter sequences. Deep RNA sequencing also contributes to the identification of sRNAs among which some were further experimentally validated. This approach, selective for the 5′ ends of primary transcripts, has been used for transcriptome analysis, TSS determination, and regulatory RNA discovery in many other pathogenic bacteria, including *Mycobacterium tuberculosis, Legionella pneumophila*, and *Pseudomonas aeruginosa* (Sahr et al., 2012; Wurtzel et al., 2012; Cortes et al., 2013). These results should improve our knowledge of gene regulatory circuits that control gene expression in this emerging zoonotic pathogen.

## Material and methods

### Strains, culture conditions, and RNA isolation

*L. interrogans* serovar Manilae strain L495 was grown aerobically at 30°C in Ellinghausen-McCullough-Johnson-Harris medium (EMJH) (Ellinghausen and McCullough, 1965) with shaking at 100 rpm to mid log phase (~1 × 10^8^ Leptospira/ml) then shifted to 37°C or maintained at 30°C for 18 h. Total RNA was extracted from triplicate cultures as previously described (Pappas and Picardeau, 2015). The quality of RNA was assessed using a Bioanalyzer system (Agilent). Ribosomal RNA was depleted by specific rRNA modified capture hybridization approach (“MicrobExpress” kit, AM1905, Ambion), allowing an enrichment of messenger RNA (mRNA).

### Construction of cDNA libraries for Illumina sequencing

rRNA depleted RNA samples from triplicate exponential cultures for each of the studied temperatures (30 and 37°C) were pooled and divided into four similar fractions.

Directional cDNA libraries for whole-transcriptome sequencing were constructed by using the TruSeq Stranded RNA LT Sample Prep kit (Illumina) from enriched non-rRNAs that were fragmented by using a Fragmentation kit from Ambion, and purified on RNeasy MinElute columns (Qiagen). Fragments of cDNA of 150 bp were purified from each library and quality was confirmed on a Bioanalyzer apparatus (Agilent).

To discriminate the primary transcripts from those with processed 5′ ends for TSS mapping, the enriched non-rRNAs was (1) untreated or (2) treated with Terminator 5′ Phosphatase Dependent Exonuclease (TEX) (Epicentre), or (3) treated with TEX and then treated with tobacco acid pyrophosphatase (TAP). cDNA librairies were prepared as described for the RNA-sequencing analysis but omitting the RNA size-fractionation step. First-strand cDNA synthesis was performed by ligation with an excess of 5′ adapter (Illumina TruSeq Small RNA kit) and by reverse transcription using a random primer (RPO primer: 5′-CCTTGGCACCCGAGAATTCCANNNNNN-3′). The cDNAs were size-fractioned within the range of 120 to 250 bp on agarose gels and purified using a QIAquick Gel Extraction Kit (Qiagen). The resulting cDNAs were PCR amplified for 14 cycles using the Illumina primer RP1, and one of the indexed primers (Illumina TruSeq Small RNA kit). The resulting PCR products were purified with Agencourt AMPure Beads XP (Beckman).

Quality of the eight cDNA libraries were confirmed on a Bioanalyser (Agilent) and each library was sequenced in single-end mode for 51 bp, using an Illumina HiSeq2500 instrument (Illumina). Reads were cleaned from adapter sequences with AlienTrimmer (Criscuolo and Brisse, 2013) (version 0.4.0) and duplicates and low quality reads using PRINSEQ (Schmieder and Edwards, 2011) (version 0.20.3). The reads were aligned to the reference genome of *L. interrogans* serovar Manilae strain L495 (total genome size of 4,614,703 bases, GC% of 34.99, number of contigs is 88, and 4261 annotated coding sequences) downloaded from MaGe platform (Vallenet et al., 2013). The alignment was performed by Rockhopper software (McClure et al., 2013), allowing 5% of read length mismatches, and using 35% of read length as minimal seed. The produced alignments were filtered to remove data with 0 scores, sorted and indexed with SAMTools (Li et al., 2009). Coverage graphs representing the numbers of mapped reads per nucleotide were generated based on the sorted reads using BEDTools (Li et al., 2009; Quinlan, 2014). On each coverage graph the upper quartile normalization (Bullard et al., 2010) was performed. To restore the original data range, each graph was then multiplied by the median of upper quartiles of all graphs corresponding to the selected temperature.

After quality trimming and duplicate removal, the TSS libraries yielded a total of 1,805,824 (out of which 1,444,131 mapped) sequence reads for the 30-TEX(−)TAP(−) library, 2,128,271 (out of which 1,689,819 mapped) sequence reads for the 30-TEX(−)TAP(+) library, 1,209,046 (out of which 986,071 mapped) sequence reads for the 30-TEX(+)TAP(+) library, 1,767,042 (out of which 1,262,780 mapped) sequence reads for the 37-TEX(−)TAP(−),1,720,339 (out of which 1,169,801 mapped) sequence reads for the 37-TEX(−)TAP(+) library, and 1,010,887 (out of which 761,737 mapped) sequence reads for the 37-TEX(+)TAP(+) library. The RNA-seq librairies yielded a total of 1,256,867 (out of which 1,150,740 mapped) and 1,495,434 (out of which 1,371,362 mapped) sequence reads at 30 and 37°C, respectively, after quality trimming and duplicates removal. The amount of reads mapping to rRNA were <1% for TSS libraries and ranged between 7 and 11% for the RNA-seq libraries.

### TSS identification and classification

TSS were identified independently from differential RNA-sequencing (dRNA-seq) data of cultures grown at 30 and 37°C. Potential TSS were identified at the positions where all of the following conditions were met [eL(i) is the coverage at position i in the graph L]:

- e_minusTEX(−)TAP(+)_(i) ≥ threshold (average of 3rd percentiles of normalized TEX(+)TAP(+), TEX(−)TAP(−), TEX(−)TAP(+) graphs)
- Coverage change e_TEX(+)TAP(+)_(i) − e_TEX(+)TAP(+)_(i−1) ≥ threshold (same as above)
- Factor of coverage change: e_TEX(+)TAP(+)_(i) / e_TEX(+)TAP(+)_(i−1) ≥ threshold (1.5)
- Enrichment factor: e_TEX(+)TAP(+)_(i) / e_TEX(−)TAP(−)_(i) ≥ threshold (1.5).

TSS candidates within five nts from each other were clustered together, and in each cluster a TSS with the strongest coverage in TEX(+)TAP(+) graph was selected as the representative TSS.

Following Dugar et al. (2013), each TSS was classified as a gene TSS (gTSS), an internal TSS (iTSS), an antisense TSS (asTSS), or an orphan (oTSS) if it could not be assigned to any of the previous classes. A TSS was classified as gTSS if it was located ≤300 bp upstream of a gene. The TSS with the strongest expression values (maximum peak height) among gTSS of a gene was classified as primary (pTSS), the rest of the gTSS that were assigned to the same gene were classified as secondary TSS (sTSS). iTSS were located within an annotated gene on the sense strand and asTSS were located inside a gene or within ≤100 bp on the antisense strand. Integrative Genomics Viewer (IGV) (Robinson et al., 2011) was used to visualize the reads and location of TSS.

The clusters of orthologous groups (COG) (Tatusov, 1997) annotations of the mRNA of *L. interrogans* serovar Manilae strain L495 are available on the MaGe platform (Vallenet et al., 2013). We compared the distribution of COG classes in leaderless mRNA (whose pTSS are located between 0 and 10 nts) in comparison to genome-wide expected probabilities. To calculate the significance of leaderlessness for each COG category the Fisher exact test was used [SciPy library (Oliphant, 2007) for Python] with the following data: in the contingency table, the genes with a detected pTSS were divided into leaderless and others on the one hand, and those that belong to the selected COG category and belong to another category on the other hand. The null hypothesis was that leaderless and non-leaderless genes are equally likely to belong to the selected COG category. A *P* ≤ 0.05 indicated strong evidence against the null hypothesis.

### Motif detection in promoter sequences

For the genes with pTSS and a 5′UTR of at least 6 nucleotides, we looked for Shine-Dalgarno (SD) sequences upstream of the start codon. Following the procedure described by Noguchi et al. (2008), we considered the nine hexamers derived from the sequence G(A/T)(A/T)AGGAGGT(G/A)ATC (complementary to a tail of 16S rRNA) as the potential SD motifs. For each gene we selected the upstream region of the start codon of up to 30 nucleotides long and looked for perfect matches or for 1-base mismatch of these nine motifs. Using the detected sequences, we constructed a position weight matrix (PWM) for each motif. Then for each gene we selected a sequence with the highest score $max_{m,j}\;[\omega_{m}\;\times \;\sum_{i\;=\;1}^{6}log\;\left(\frac{p_{m}\left(x_{i,j}\right)}{q\left(x_{i,j}\right)}\right)]$, where ω_*m*_ is a frequency of a motif *m*,*x*_*i, j*_ is an *i*^th^ nucleotide of a hexamer *j, p*_*m*_(*x*_*i, j*_) is a frequency of *x*_*i, j*_ at a position *i* of a PWM for a motif *m*, and *q*(*x*_*i, j*_) is a background frequency of *x*_*i, j*_ calculated from a GC content of the genome.

We extracted the 80 nucleotides upstream of the identified pTSS in *L. interrogans*, and performed motif discovery in these sequences using the MEME algorithm implemented in the MEME suite version 4.10.1 (Bailey et al., 2009). We looked for motifs of minimal length five that occur zero or one time per sequence, and are found in at leasts two sequences. We then compared motifs against the Swiss Regulon *Escherichia coli* motifs database (Pachkov et al., 2013), and against CollecTF, a database of transcription factor binding sites (TFBS) in the Bacteria domain (Kiliç et al., 2014). The comparison was performed with Tomtom, a motif comparison tool (Gupta et al., 2007) from the MEME suite. We have also scanned for the bacterial TFBS motifs and for the *E. coli* regulatory motifs in the 80 nucleotides upstream of the identified *L. interrogans* pTSS, using FIMO tool (Gupta et al., 2007; Grant et al., 2011) from the MEME suite.

For the prediction of promoter sequences for the housekeeping sigma factors sigma70, and alternative sigma factors sigma28 and sigma24, the following matrix and spacer between −35 and −10 (in parenthesis) were used: Matrix.18_15_13_2_1.5 (13–19) for sigma70, Matrix.15.13.11.8.5.d.NC (13–15) for sigma28, and Matrix.15.13.15.5.8.d.NC (15–20) for sigma 24 (matrix resource: http://www.ccg.unam.mx/Computational_Genomics/PromoterTools/). The 80 nucleotide sequences upstream TSSs were then subjected to prediction of the presence of each sigma factor-type promoter sequence using PromoterHunter software (http://www.phisite.org/main/index.php?nav=tools&nav_sel=hunter). For the prediction of sigma54 promoter sequences, the PATSER program (Hertz and Stormo, 1999) was used to search against 80 nucleotide sequences upstream of the identified pTSS. The weight matrix of the −24/−12 sigma54-type promoter consensus sequence used in this study was based on a set of 186 RpoN-dependent promoters from different bacterial species (Barrios et al., 1999). The actual scores for the sequences were determined from the weight matrix. The higher the score, the higher the specificity. A cutoff of score >4.0 was chosen as a potential sigma54 promoter.

### Operon prediction

Operon detection was performed using software Rockhopper (McClure et al., 2013) on the total RNAseq data at 30 and 37°C. Rockhopper detects operons using a naive Bayes classifier based on prior operon probabilities, intergenic distance, and correlation of gene expression across RNA-seq experiments. Potential pTSS was identified for each operon as the pTSS detected on dRNA-seq data (see above) for the first gene of the operon. For operons with no pTSS detected on dRNA-seq data, the value identified by Rockhopper on the total RNAseq data (in the majority of cases equal to the start of the first operon gene) was used.

### Putative sRNA prediction

Putative sRNA detection was performed using software Rockhopper (McClure et al., 2013) on the total RNAseq data at 30 and 37°C. Among the transcripts identified by Rockhopper as predicted RNA, those of the length ≥50 nucleotides were kept. For each sRNA, potential pTSS were identified following the procedure described above, and potential small coding sequences were detected using any of the start codons ATG, TTG, GTG, and the stop codons TAA, TAG, TGA. For each putative sRNA, a search for matching families in Rfam database (Nawrocki et al., 2015) was performed via RESTful interface using urllib2 library for Python.

The secondary structure was predicted for each putative sRNA sequence using UNAFold (Markham and Zuker, 2008; version 3.8). Rho-independent terminator (RIT) sites were detected at positions −25 to 200 nucleotides of stop codon of each putative sRNA using Arnold software (Naville et al., 2011). We filtered out the RIT sites with values of Gibbs free energy of more than −4 kcal/mol. Putative sRNA were classified into the following categories: antisense CDS (sRNA located on an opposite strand to a coding sequence), antisense 5′UTR (sRNA located on an opposite strand to the 5′UTR of a coding sequence), antisense 3′UTR (sRNA located on an opposite strand to the 3′UTR of a coding sequence), and IGR (sRNA located in an intergenic region). Manual inspection and curation of sRNA was performed with IGV.

### 5′-RACE

*L. interrogans* total RNA was prepared from cultures grown in EMJH at 30°C at exponential growth as previously described (Pappas and Picardeau, 2015) and subjected to 5′ rapid amplification of cDNA ends (RACE) with the 5′ RACE system from Invitrogen, according to the manufacturer's instructions. The gene-specific primers for reverse transcription reactions and generation of 5′ RACE amplicons are listed in Supplementary Table 1. PCR products were then cloned in pCR2.1-TOPO (In vitrogen) and plasmid DNA was isolated from 5 ml of overnight culture of *E. coli* using Qiagen miniprep kit (Qiagen). Plasmids were then sequenced by Eurofins.

### Northern blot

To confirm the expression and size of putative sRNA, 2 μg of total RNA extracted from *L. interrogans* serovar Manilae were mixed together with one volume of denaturing loading buffer containing 95% formamide (Thermo Fisher), incubated at 95°C for 5 min and then placed on ice. Samples were separated by 8 M urea polyacrylamide gel (concentration ranging from 5 to 10%) in TBE buffer, along with an RNA ladder (Euromedex), for 1 h at 25 mA. The RNA integrity of samples following migration was evaluated by ethidium bromide staining (0.5 μg/mL). Gels were then transferred onto Hybond N+ membranes (Amersham) using a Criterion Blotter in TBE buffer for 1 h at 50 V. RNA molecules were crosslinked to the membranes by UV irradiation (0.51 J/cm^2^) and pre-hybridized with 10 mL of ULTRAhyb hybridization buffer (Thermo Fisher) for 1 h at 42°C in a rotating chamber; then, 2 μL of 10 μM 5′biotinylated oligo DNA probe (Supplementary Table 2) were added and hybridization proceeded for 14 h. Membranes were washed twice in 2X SSC and 0.1% SDS and then twice in 0.1X SSC and 0.1% SDS. Hybridized probes were visualized by incubation with horseradish peroxidase-conjugated streptavidin and chemiluminescent substrate (Thermo Fisher), followed by film exposure.

### Availability of supporting data

The raw data files for the RNA-seq experiment are deposited in the Gene Expression Omnibus (GEO) database from NCBI (Edgar et al., 2002), Gene accession GSE92976. Additionally, the genome files of *L. interrogans* serovar Manilae strain L495 used for analysis of RNA-seq data are available in MicroScope (http://www.genoscope.cns.fr/agc/microscope/home/index.php).

## Results

To obtain an overview of the *L. interrogans* transcriptome, the pathogen was grown at 30°C for optimal *in vitro* growth and at 37°C to mimic the host environment and to promote the expression of genes important during the infection.

RNA-seq data of the most abundant transcripts showed that lipoproteins-encoding genes *lipL32, lipL21, lipL41, loa22*, and *lipL36*, 30S and 50S ribosomal subunit proteins-encoding genes, and flagellin-encoding genes were the most highly expressed genes in *L. interrogans*, which concurs with previous transcriptional and translational analyses (Lo et al., 2006; Malmström et al., 2009). Additionally, heat shock protein-encoding genes *groS* (LMANv2_150128), *groEL* (LMANv2_150129), *hsp15* (LMANv2_380017), and *hsp15*-like (LMANv2_380018) were up-regulated (two- to three-fold increase in transcript levels) by temperature upshift (Supplementary Table 3). Together, these results indicate that RNA preparations and temperature shift experiments were performed in a manner acceptable for subsequent transcriptome analysis. Interestingly, a 92-nucleotide gene (LMANv2_330026) was the second most highly expressed gene after *lipL32* at both 30 and 37°C. The conservation of this small gene in all leptospiral species suggests that it may play an important role in leptospiral physiology.

### TSS mapping

The vast majority of mRNAs are synthesized with a 5′-triphosphate group (5′ PPP), while the 5′ ends of transcripts generated through RNA processing and degradation, have a monophosphate group (5′ P) (Wurtzel et al., 2010). For TSS mapping, three libraries were carried out for each biological sample: one library was generated from RNA treated with terminator 5′ phosphate dependent exonuclease (TEX), which specifically degrades RNA species that carry a 5′ P, then enriching for transcripts that carry a 5′-PPP. A second library was generated from untreated total RNA. In the third library, the exonuclease-resistant RNA (primary transcripts with 5′PPP) was treated with TAP, which degrades 5′ PPP to 5′ P, making them accessible for 5′ end linker ligation. Comparing these libraries enables determination of putative TSSs (see Material and Methods). An increased number of sequencing reads from a 5′ end following TAP treatment is an identifier of a TSS.

**Figure 1:**
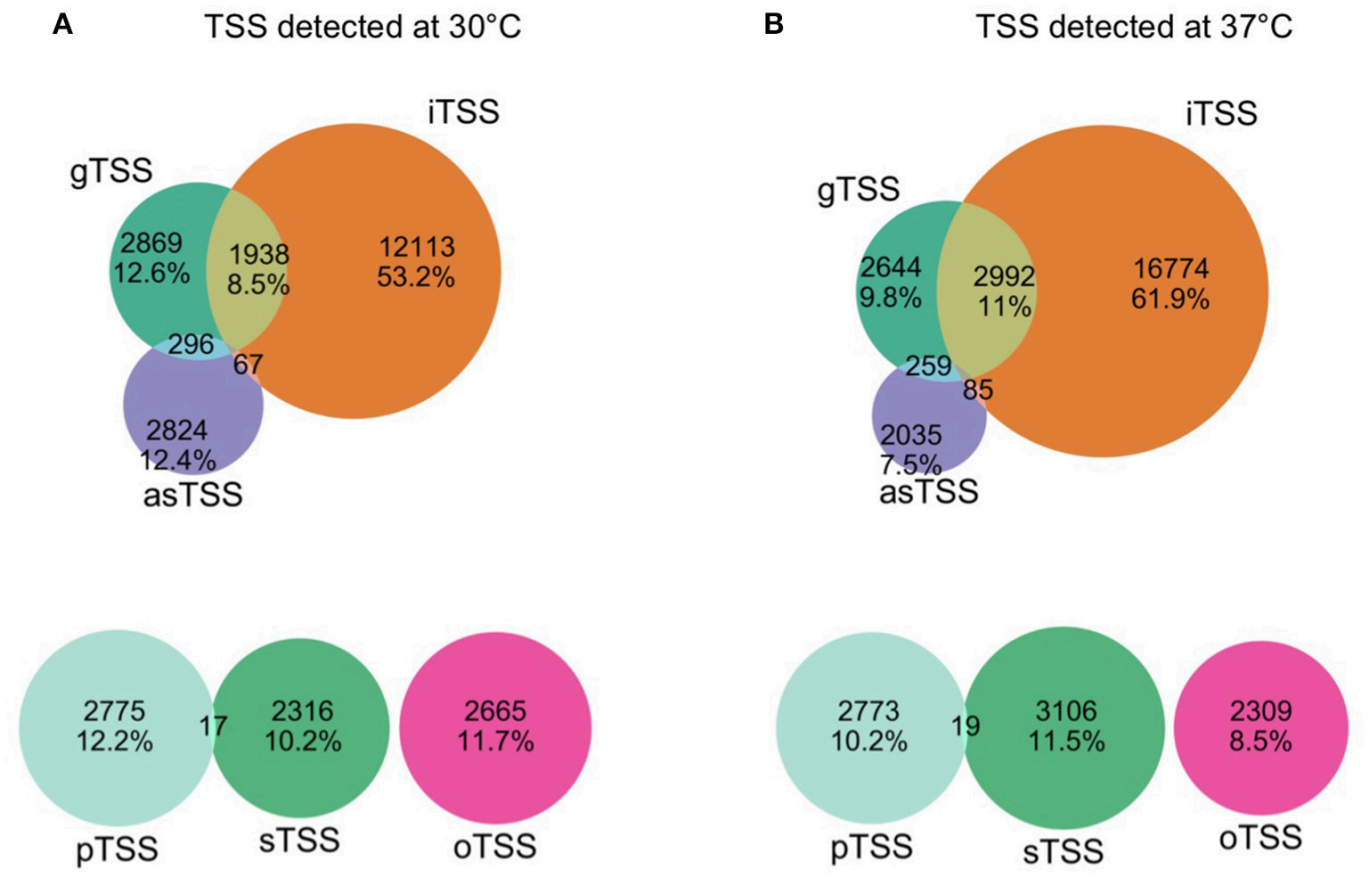
**Venn Diagram of TSS detected at 30°C (A)** and 37°C **(B)**. TSS were classified as gene TSS (gTSS), internal TSS (iTSS), antisense TSS (asTSS), or orphan (oTSS) (see Material and Methods). The TSS with the strongest expression values (maximum peak height) among gTSS of a gene was classified as primary (pTSS), the rest of the gTSS that were assigned to the same gene were classified as secondary (sTSS). TSS can be affiliated to multiple categories.

**Figure 2:**
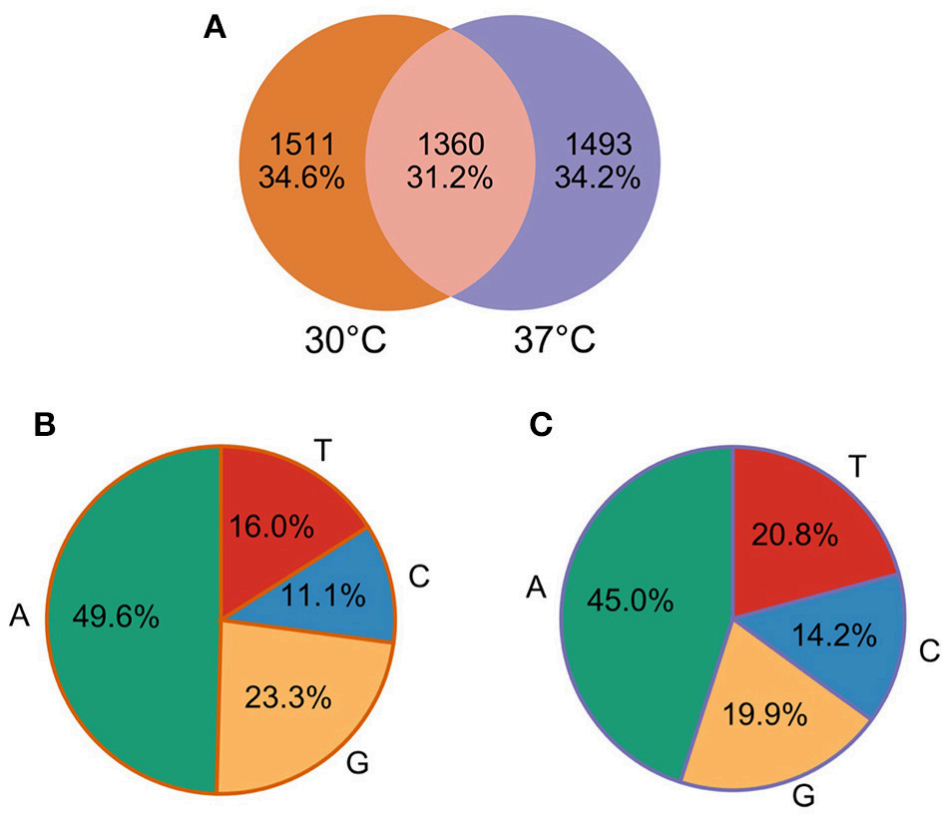
**Primary TSS (pTSS) detected at 30 and 37°C. (A)** Venn Diagram of pTSS at 30 and 37°C. **(B)** Nucleotide preference at the predicted pTSS at 30°C. **(C)** Nucleotide preference at the predicted pTSS at 37°C.

Our comparative approach enabled the annotation of a total of 25,397 and 30,739 TSS at 30 and 37°C, respectively. TSSs were classified into different categories: gene TSS (gTSS), including primary TSS (pTSS) and secondary TSS (sTSS), internal TSSs (iTSS), including antisense TSSs (asTSS), and orphan TSSs that do not belong to the other categories (Figure 1). The genome position of all TSSs detected at 30 and 37°C is listed along with their categorization as primary, secondary, antisense, internal, or orphan TSS (Supplementary Table 4). Notably, one TSS can independently be assigned to more than one category. For example, within operon-like structures the pTSS of the downstream gene can also be internal to the upstream gene. In total, 2865 and 2866 pTSS of annotated genes or operons were identified in the genome of *L. interrogans* at 30 and 37°C, respectively. A total of 2437 and 3214 sTSS, defined as a TSS being located in close proximity of a pTSS but having fewer reads, were also detected at 30 and 37°C, respectively (Supplementary Table 4). Genes that were not assigned a TSS may be organized into operons (see below) or were not expressed at detectable levels. Thus, 72 and 87% of genes detected by RNA-seq at 30 and 37°C possess a pTSS, respecitvely, while only 17 and 43% of non-expressed genes at 30 and 37°Cwere assigned a pTSS, respectively. Approximately 22.6% of the pTSS identified are conserved at 30 and 37°C. In contrast, only 5.5% of the sTSS are conserved. When grouping together pTSS with a position within a distance of five nucleotides (±5 nt), 1360 pTSS are conserved at 30 and 37°C, thus 47.22% of the pTSS at 30°C are also found as pTSS at 37°C (Figure 2A).

Sequence analysis of the nucleotide composition of pTSS revealed a strong selection of the purines A (45–50%) and, to a lower extent, G (20–23%) at the +1 site (Figures 2B,C), which is usually required for efficient transcription initiation by RNA polymerase.

**Figure 3:**
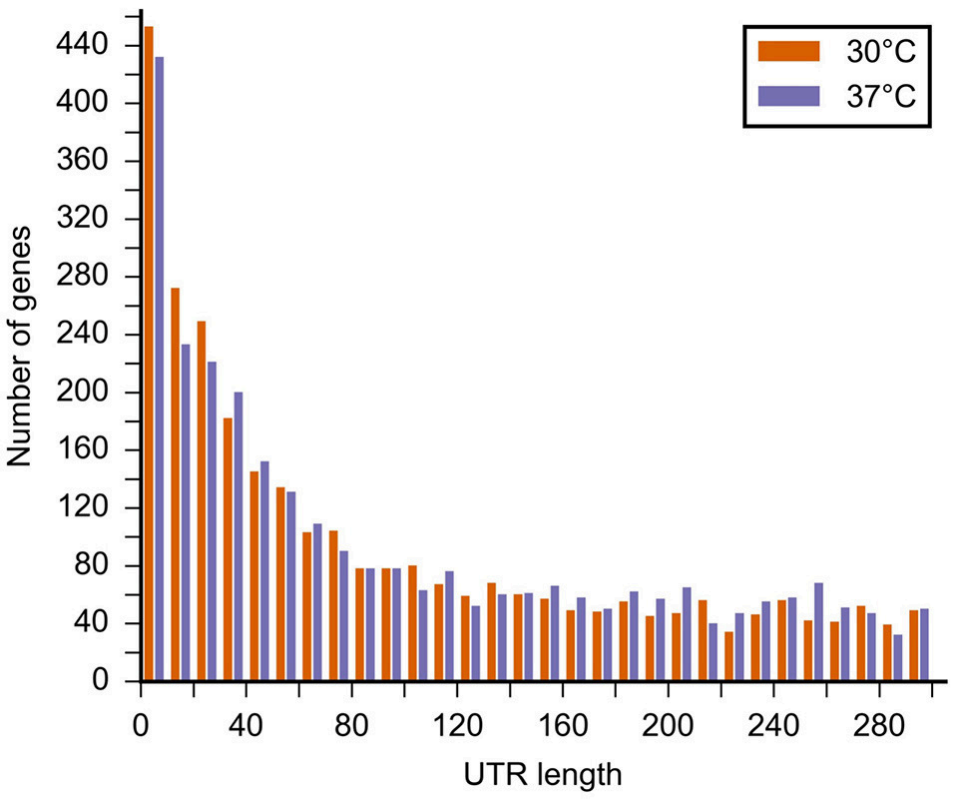
**Length distribution of the 5′UTR of the mapped pTSS at 30 and 37°C in the *L. interrogans* genome**. The graph shows the length of the 5′UTR (distance from the predicted translational start to the TSS).

We analyzed the length distribution of the 5'UTR of the genes for which the pTSS were detected (Figure 3). We found a median 5′ UTR length of 91–97 nucleotides at 30 and 37°C, respectively. The majority of *L. interrogans* genes (430–450 genes) had a pTSS located within 10 bp of the translational start codon (Figure 3). Among those are 184 and 170 genes where the pTSS is identical to the translational start at 30 and 37°C, respectively (244 and 231 genes at 30 and 37°C, respectively, if we include pTSS at the −1 position). Considering these genes as leaderless, we analyzed the dependency between leaderlessness and COG. At both 30 and 37°C leaderless genes were underrepresented in categories C (energy production and conversion) and V (defense mechanisms), and overrepresented in category R (general function prediction only). At 30°C they were also overrepresented in H (coenzyme transport and metabolism). At 37°C leaderless genes were additionally underrepresented in N (cell motility) and overrepresented in E (amino acid transport and metabolism), F (nucleotide transport and metabolism), and G (carbohydrate transport and metabolism). In the other categories differences between representation of leaderless and leadered genes was not significant. Temperature shift did not result in any significant difference, as determined by Student's *t*-test, in the relative expression of leaderless mRNAs for specific COGs.

We selected 10 genes of known function with mapped pTSSs to verify the reliability of TSS designation by 5′ RACE experiments. There was good agreement between RACE determined and predicted TSS positions, with a maximum divergence of three nucleotides, except for one gene, *ahpC*, for which the TSS determined by RACE is located 17 nucleotides downstream from the predicted TSS (Table 1). We also compared our data with TSSs experimentally mapped in previous studies. The TSSs identified in *ligA* (Matsunaga et al., 2013), *groS*, and *groEL* (Ballard et al., 1993) were re-confirmed in this study, providing further validation of our TSS mapping (Table 1).

**Table 1:** **Comparison of *L. interrogans* TSS identified by RNA-seq with TSS identified by 5′RACE**.

**Gene**			**Distance of TSS from CDS^a^**	
			**RNAseq**	**5′ RACE**
LMANv2_60079	*flgB*	Flagellar basal body rod protein FlgB	13	12
LMANv2_110011	*dapA*	4-Hydroxy-tetrahydrodipicolinate synthase	0	0
LMANv2_150128	*groES*	Chaperone Hsp10	56	58^b^
LMANv2_370081	*fumC*	Fumarate hydratase	40	40
LMANv2_580002	*ahpC*	Peroxiredoxin	40	23
LMANv2_280031	*perR*	Ferric uptake regulator-like	1	0
LMANv2_680004	*hemO*	Heme oxygenase	22	21
LMANv2_160018	*mreB*	Actin-like component MreB	86	84
LMANv2_150111	*lipL32*	Lipoprotein LipL32	17	18
LMANv2_150129	*groEL*	Chaperone Hsp60	170	167^c^
LMANv2_630002	*ligA*	Immunoglobin-like repeats LigA	176	175^d^
LMANv2_460028	*hfq*	RNA-binding protein Hfq	146	146

a *Position 0 corresponds to the first nucleotide of the start codon*.

b *Previously identified in L. interrogans serovar Copenhageni by primer extension, see Ballard et al. (1993)*.

c *TSS previously identified at position 61 in L. interrogans serovar Copenhageni by primer extension, see Ballard et al. (1993)*.

d *Previously identified in L. interrogans serovar Copenhageni by 5′-RACE (Matsunaga et al., 2013)*.

### Operons

We defined operons in the *L. interrogans* genome as regions with continuous coverage of whole transcript reads by RNA-seq and the presence of a pTSS in the upstream sequence of coding sequences. Using these criteria 750 operons of 2–19 genes (for a total of 2181 genes) were defined at both 30 and 37°C (Supplementary Table 4). The average operon size of *L. interrogans* was 2.9 genes. The largest operon was 17 kb long and codes for enzymes of amino acid and cell biosynthetic pathways (*dapA-dapB-rpsB-trpA-trpB-pyrH-uppS-proS*). The second largest operon contained 16 genes (*cbiX-cbiD-cbiC-cbiT-cobI-cobJ-cobM-cobB-cobU-cobDQ-cobD*) which are involved in vitamin B12 biosynthesis. Other large operons include phage-related genes (13 genes, including genes encoding base-plate J-like and tail fiber domain proteins), and genes coding for a type II secretion system (13 genes including *gspC-gspD-gspE-gspF-gspG-gspH-gspJ-gspK-ftsA*), sialic acid biosynthesis (12 genes including *neuA1-rfb3-neuB-neuC-neuD-neuB2-neuA2*), and NADH dehydrogenase complex 1 biosynthesis (12 genes *nuoA-nuoB-nuoC-nuoD-nuoE-nuoF-nuoH-nuoK-nuoN*). The *L. interrogans* genome contains about 50 genes involved in the synthesis of the endoflagellum. Most of these genes (71%) are organized in 8 operons (from 2 to 7 genes).

For most of the downstream genes within operons, a pTSS can also be internal to the upstream genes, suggesting that the operon's genes can be transcribed through alternate promoters.

### Motifs in promoter regions

Shine-Dalgarno sequences are defined as purine-rich hexamers complementary to the 3′-end of the 16S rRNA between 1 and 40 bp upstream of an annotated start codon. Approximately 70% of the genes with a pTSS had a predicted Shine-Dalgarno motif (Supplementary Table 5).

We aligned the upstream sequences of all identified pTSSs (−80 to +1) by MEME to identify potential sequence motifs in promoter regions. This resulted in the detection of two distinct sequence motifs with *P*-values below e-10 at both 30 and 37°C. These two conserved motifs, [TA]A[TA]TAGA[AG]TTGTTGAAAAATTAATTCTCCAT[CT][TG][GA]TTTC[TC]ATTT[TC]A and TGT[AG]G[GT]A[AG][TC]T[CA]C[CT]ACA[AT][AT][TA][TAC], (i) do not have a specific nucleotide position relative to the TSS, (ii) do not resemble motifs and TFBS from the *E. coli* database, (iii) are part, at least most of them, of an intergenic repeated element, and (iv) are not found in the promoter region of the expressed gene as identified by RNA-seq (our study) and by mass spectrometry (Malmström et al., 2009). Taken together, these results suggest that these motifs may not represent DNA-binding sites (Supplementary Table 6).

### Sigma factors

The *L. interrogans* genome is predicted to contain 4 sigma factors: the housekeeping sigma factor σ70 (RpoD) and the alternative sigma factors σ28 (RpoF), σ54 (RpoN), and σ24 (RpoE) which provide promoter recognition specificity for the polymerase and contribute to environmental adaptation of the bacterium. We performed an *in silico* genome-wide search for putative σ70, σ28, and σ24-type promoters. The matrices used were derived from different *E. coli* promoter sequences. Given that *L. interrogans* has an AT-*rich* genome, we selected stringent criteria (see Material and Methods). We performed an *in silico* genome-wide search for putative σ70 and σ54-binding sites. A σ70-like promoter sequence (TTGACA <16–18 bp>TATAAT in *E. coli*) is found in more than 1000 *L. interrogans* genes at both 30 and 37°C (Supplementary Table 7). However, our analyses may fail to accurately predict this promoter sequence in the AT-*rich L. interrogans* genome and most of the identified promoter sequences most likely do not operate as σ70-binding sites. The σ54 recognizes a unique −24/−12 promoter sequence (CTGGNA <6 bp>TTGCA in *E. coli)* and is activated by enhancer-binding protein (EBP). *L. interrogans* contains two EBPs, EBP-A and EBP-B. Each EBP-σ54 pairs may respond to different signals to activate distinct transcripts of genes. A typical σ54-binding site was identified in the promoter regions of three genes encoding for putative lipoproteins (LMANv2_200027/LIC12503 and LMANv2_290065/LIC11935) and the ammonium transporter AmtB (LMANv2_310003/LIC10441) at both 30 and 37°C (Supplementary Table 8). Our previous EMSA results show that both recombinant σ54 and EbpA proteins are able to bind a 50-bp oligonucleotide encoding the predicted −24/−12 promoter regions of these three genes, indicating that the σ54-binding motif of *L. interrogans*, [TA][TG[CG][TAC]A <6 bp>T[GT][GC]CA, closely resembles the *E. coli* motif (Hu et al., 2017). The alternative sigma factor σ28 (sigma F) is known to regulate flagellar genes in most bacteria and predicted σ28-binding sites at position −35 and −10 from the TSS in *L. interrogans* promoter sequences comprise at least four genes coding for components of the endoflagellum (LMANv2_260046/FlaA1, LMANv2_290016/FlaB1, LMANv2_590023/FlaB4) and the flagellin-specific chaperone FliS (LMANv2_10030). Previous works have shown that σ24 (*rpoN*) is necessary for resistance to heat shock and other environmental stresses in bacteria. 469 putative σ24 binding sites are detected in the promoter regions of *L. interrogans* at both 30 and 37°C (Supplementary Table 8). However, σ24 promoter sequences have a −35 region less well-conserved in phylogenetically distant bacteria, hence making prediction of binding sites in *L. interrogans* challenging.

### Identification of small non coding RNA (sRNA)

**Figure 4:**
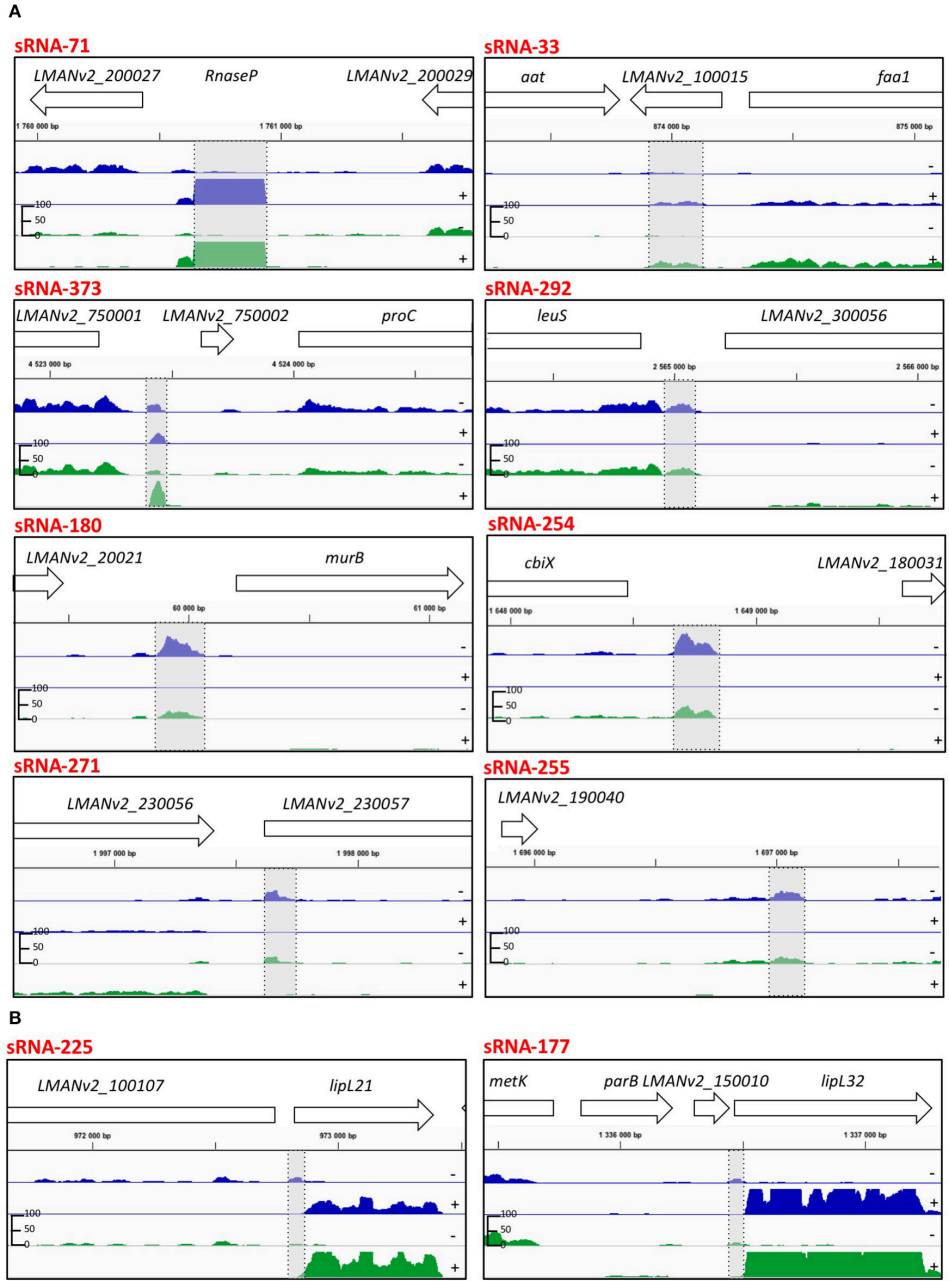
**IGB viewer of representative predicted sRNAs in the *L. interrogans* genome. (A)** Candidate sRNAs that were validated by Northern blot (Figure 5). **(B)** Candidate sRNAs for *lipL21* and *lipL32*. Visualization of normalized mapped reads for minus (−) and plus (+) strand. Blue and green reads indicate mapped reads at 30°C and 37°C, respectively. The vertical “read count” scale is 0–100. Genomic locations and CDS are also indicated. Highlighted in gray is the predicted sRNA.

sRNAs are usually defined by their position in the genome relative to their target genes, with *cis*-encoded sRNAs located antisense to their target and *trans*-encoded sRNAs in intergenic regions of the genome away from their target. After manual curation, a total of 277 (pTSS annotated for 176) and 226 (pTSS annotated for 137) sRNAs were found in *L. interrogans* at 30 and 37°C, respectively; including 137 sRNAs that are conserved at both temperatures (Figure 4A). The predicted sRNAs displayed an average size of 101 and 98 nt at 30 and 37°C, respectively (Supplementary Table 9). The majority of predicted sRNAs, 168 and 147 at 30 and 37°C, respectively, were found to be located in the intergenic regions of the *L. interrogans* genome. We also identified a total of 98 and 75 antisense RNA (asRNA) candidates, at 30 and 37°C, respectively, which are located antisense inside coding regions. In addition, 29 and 19 asRNA candidates at 30 and 37°C, respectively, that are opposite to a 5′UTR or 3′UTR were detected (Supplementary Table 9). asRNAs overlap either with the 5′ end (14–17%), the 3′ end (9–11%), or the central region (72–77%) of the gene found on the opposite strand. The vast majority (>60%) of asRNAs overlap with genes coding hypothetical proteins; other targeted genes with a putative known function include the genes encoding lipoproteins LipL32 and LipL21 (Figure 4B), a TonB dependant receptor, a permease, and an anti-anti sigma factor (Supplementary Table 9).

Compared to the sRNA sequences in the Rfam database, few *L. interrogans* sRNAs displayed homology with well characterized sRNAs in other bacteria. Among those are a cobalamine riboswitch, tRNAs, tmRNA, also known as SsrA, RNase P RNA, and 5S rRNA. This lack of orthologs suggests these sRNAs to be novel with completely unknown function. RIT sequences were also searched at the 3′ end of the sRNAs, and 16 of the sRNAs contained typical RIT sequences, including seven that are conserved at both 30 and 37°C, indicating that the vast majority of sRNAs did not contain typical RIT (Supplementary Table 9). We scanned the sRNAs for the presence of small open reading frames. A total of 40 and 22 putative ORFs were identified at 30 and 37°C, ranging in size from 28 to 78 codons (Supplementary Table 9). The putative gene products were then examined for the presence of conserved protein domains using Blast and InterProScan. None of the deduced proteins, however, contained a known protein domain, suggesting that they may not correspond to coding regions. Secondary structures of all sRNAs were determined by minimum free energy folding and RNA shape analysis which achieved high shape probabilities in most cases (Supplementary Table 10).

**Figure 5:**
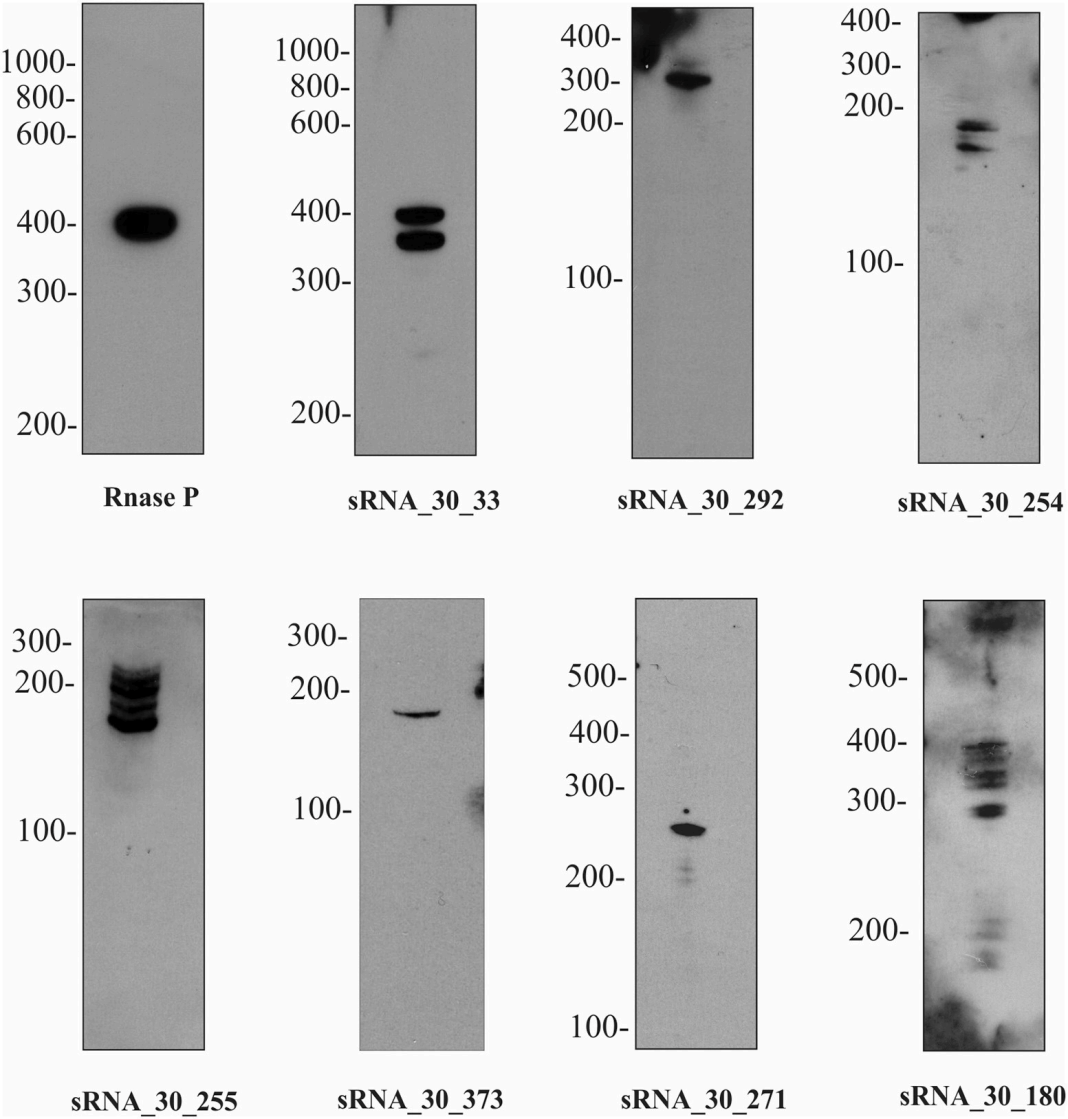
**Expression of selected sRNAs in *L. interrogans***. A subset of sRNAs identified by RNA-seq were validated by Northern blot. One representative lane was displayed for each sRNA identified. The names of sRNAs and the molecular marker were indicated on the top and on the left, respectively. For more details of each sRNA see Figure 4A.

To independently confirm the presence and size of sRNAs identified by transcriptome sequencing, Northern blotting was performed on 13 abundant sRNAs and putative sRNAs of *lipL21* and *lipL32* (Supplementary Table 2, Figures 4A,B, 5). This analysis was carried out on cells grown to exponential phase at 30°C. Use of a non-radioactive labeling method confirmed the presence of eight of the sRNAs (Figure 5). For four of those, the size estimated from the transcriptome was within the size estimated from Northern blotting. In other cases, the detected transcript exceeded the size predicted by the RNA-seq data. The discrepancy in lengths may be explained by *in silico* prediction criteria. While most sRNAs displayed single and specific bands, some sRNAs exhibited additional bands which could be due to RNA processing or alternative transcription initiation (Figure 5).

## Discussion

In 2003, *L. interrogans* serovar Lai was the first *Leptospira* genome to be sequenced (Ren et al., 2003). Today, the genome sequences of hundreds of *Leptospira* strains have been determined, including representations of each of the 20 *Leptospira* species (Fouts et al., 2016). However, the difficulty of generating mutants in pathogenic strains limited the ability to analyse the wealth of information contained in these genomes and the molecular basis of leptospiral pathogenesis remains poorly understood. In this study, a combination of TSS mapping with total RNA-seq has generated a comprehensive overview of the transcriptional landscape of the pathogen *L. interrogans*.

Promoter regions are poorly characterized in *Leptospira* spp. To date, few experimentally proven TFBS have been described (Cuñé et al., 2005; Morero et al., 2014; Hu et al., 2017) in the literature and promoter prediction algorithms and *E. coli* consensus sequences of DNA motifs are not applicable to the *Leptospira* genome. Here, we annotated 2865 and 2866 pTSSs in *L. interrogans* at 30 and 37°C, respectively. Our 5′RACE results showed that our RNA-seq analysis accurately captured the TSS, confirming the accuracy of our TSS mapping. In *L. interrogans*, the majority of 5′-UTRs appear to be <80 bp, which is common for bacteria. We identified an unexpectedly high number of leaderless mRNAs, including a significant fraction of learderless mRNAs encoding products with unknown function (COG R).

We identified ~440 leaderless mRNAs having a UTR length of <10 nucleotides, among those half of them have a UTR length of <2 nucleotides. Studies in most bacteria have typically reported only a few leaderless mRNAs as, for example, 12 leaderless genes in *L. pneumophila* (Sahr et al., 2012), 20 in *Helicobacter pylori* (Bischler et al., 2015), 23 in *Salmonella typhimurium* (Kröger et al., 2012), 30–41 in *Prochlorococcus* spp. (Voigt et al., 2014), and 57 in *Bacillus amyloliquefaciens* (Liao et al., 2015). However, an abundance of leaderless transcripts have recently been identified in the genomes of *Deinococus deserti* (1174 leaderless mRNAs) (de Groot et al., 2014), and *M. tuberculosis* (505 leaderless mRNAs) (Cortes et al., 2013). Translation of leaderless transcripts may influence translation efficiency in certain conditions and/or the half-life of trancripts (Cortes et al., 2013).

We extracted the sequences upstream of the identified TSSs and analyzed them for common motifs. This approach identified highly conserved RpoN (σ54) promoter elements. However, other motifs were not identified in the promoter regions, emphasizing the relative lack of highly represented promoter motifs for *L. interrogans* transcripts. This may be due to (i) the low G+C content (35%) of the *L. interrogans* genome, (ii) the variability of the distances from the position relative to the downstream pTSS (−24/−12 or −35/−10 promoter sequences) making the identification of consensus sequences difficult, (iii) the multitude of different promoter sequences that are recognized by a variety of sigma factors and other transcriptional regulators, (iv) the inaccuracy of TSS mapping, or (v) the difficulty of predicting the *L. interrogans* promoter motifs based on *E. coli* consensus sequences. These results have significant implications for understanding the structure of promoters in *L. interrogans*. However, experimental identification of regulatory regions is necessary to improve the prediction of possible binding sites as well as to identify sequence properties that distinguish between active and weak/inactive promoters.

The genetic manipulation of pathogenic leptospires remains challenging due to its poor transformation efficiency and/or its inefficient homologous recombination machinery (Picardeau, 2015). We recently described a new strategy for creating targeted gene knockdowns in both saprophytic and pathogenic *Leptospira* spp. using TALE (Transcription Activator-Like Effector) system (Pappas and Picardeau, 2015). Since the role of a TALE protein is to repress transcription by binding directly to DNA within the promoter region of a gene (which in turn inhibits promoter recognition by RNA polymerase or by abrogating transcription initiation), identification of TSS from this study will prove helpful for designing TALEs for targeted genes in the future.

iTSSs are the most abundant category of TSSs identified by this study. The majority of operons in *L. interrogans* are complex with internal promoters overlapping other genes that may generate multiple transcription units. Presence of iTSSs may also be due to incorrect start codon annotation or may be the result of processed derivatives of longer mRNAs (Schlüter et al., 2013). Even though a direct comparison with other TSS identification studies is not possible, it is worth mentioning that similar to *L. interrogans*, a high percentage of internal TSSs were also observed in *Borrelia burgdorferi* (Adams et al., 2016). Further experimental analysis is therefore required to validate the presence of these iTSSs.

*L. interrogans* has also a number of oTSSs (2636 and 2278 at 30 and 37°C, respectively, not associated with any CDS) similar to the number of pTSS. These oTSS may correspond to putative novel CDSs or sRNAs. oTSSs may also originate from missing gene information in the contig boundaries of the draft genome (see below).

We present here the first operon map of an *L. interrogans* genome paving the way to a full understanding of the complex transcriptional regulations governing the life cycle of this pathogen. A total of 750 multi-gene operons were predicted in *L. interrogans* that were mostly composed of two (57%) or three (23%) genes, as well as 10 operons that included more than ten genes (1%). These co-regulated and co-transcribed genes may allow a rapid adaptation to environmental changes, and warrant further study. *L. interrogans* contains, for example, eight operons coding for the biosynthesis of the endoflagellum. The control of expression of these genes has not been investigated in *Leptospira* spp., but in other bacteria, the genetic organization into large complex units enables a tight regulation of gene expression in a cascade that closely parallels the assembly hierarchy of the flagellar structure.

Transcriptome analysis of *L. interrogans* maintained at 30°C compared to those shifted to 37°C had a relatively minor effect on TSS and sRNA mapping. Although an increase in temperature appears to be an important signal for changes in *Leptospira* gene expression, previous studies showed that other factors such as osmolarity, iron levels, and serum exposure are also important environmental signals (Adler et al., 2011). The temperature upshift from 30 to 37°C may therefore partially mimic transfer to a mammalian species.

The use of a reference draft genome with 88 contigs can lead to mapping artifacts or missing information. Gaps represent missing genomic information and, in many cases, these gaps can coincide with genes or operons that are then disregarded in genome mapping. Use of a complete reference genome will allow a more detailed analyses of our data. Our results will also allow re-annotation of the genome by the identification of novel genes and correcting mis-annotated start codons.

sRNAs typically function by binding near the translation start site of their target mRNAs and thereby inhibit or activate translation. According to the locations of sRNA genes and their targets, sRNAs can be classified into *cis*-encoded sRNAs and *trans*-encoded sRNAs. For the *cis*-encoded sRNAs, sRNA genes overlap with their target genes. Cis-encoded regulatory RNAs are sequences overlapping with their target mRNAs that are able to change their conformation in response to an environmental cue. sRNA have been reported in the genomes of *L. biflexa, L. interrogans*, and *Leptospira licerasiae* (Ricaldi et al., 2012; Caimano et al., 2014; Iraola et al., 2016). An RNA thermometer, whose structure is sensitive to temperature shifts, has been shown to be responsible for the regulation of *ligA* and *ligB* expression in *L. interrogans* (Matsunaga et al., 2013). A variety of riboswitches may also operate as intracellular sensors by binding to small metabolites or ions. Cobalamin and thiamine pyrophoshate riboswitches have been previously reported in *L. interrogans* and *L. licerasiae* (Ricaldi et al., 2012; Caimano et al., 2014; Iraola et al., 2016). Binding of the effector molecule influences the secondary structure of the riboswitch part of the mRNA, which in turn affects gene expression. A previous transcriptome study of *L. interrogans* serovar Copenhageni within the mammalian host identified 11 sRNAs, which were confirmed by qRT-PCR (Caimano et al., 2014). Most of the 11 sRNAs identified in *L. interrogans* serovar Copenhageni are conserved in *L. interrogans* serovar Manilae in this study. Thus LIC2nc40, LIC2nc10 (cobalamin riboswitch), LICnc60 (RNase P), and LICnc10 (tmRNA) were also detected in our study. Other previously described sRNAs (LIC1nc80, LIC2nc20, LIC1nc11) are annotated as protein coding genes in *L. interrogans* serovar Manilae and may encode small proteins. In this study, we identified 277 and 226 putative sRNAs in *L. interrogans* serovar Manilae at 30 and 37°C, respectively, suggesting that a substantial number are novel sRNAs candidates. The relatively high number of sRNAs found in our study is likely due to differences in regards to library preparation strategies and the dRNA-seq approach used. A recent study in *B. burgdorferi*, which is one-third the size of the genome of *L. interrogans*, identified 351 putative sRNAs (Arnold et al., 2016), suggesting that spirochetes transcribe numerous noncoding RNAs which are harnessed to control transcriptional and post-transcriptional processes.

While several sRNAs have been detected in *Leptospira* spp. none had previously been experimentally validated by Northern blot. In this study, eight sRNAs were detected by Northern blotting (out of 13 tested abundant sRNAs) (Figures 4A, 5).

The majority of sRNAs (>60%) are located in the intergenic regions, separated from their target genes and may act as antisense regulators on *trans*-encoded mRNAs. However, imperfect base pairing regions within their target genes makes target gene prediction challenging. We also identified cis-endoded sRNAs which are located antisense to coding regions. Notably, expression of two *cis*-encoded sRNAs may act as antisense sRNA by base pairing at the ribosome binding site (RBS) region of *lipL21* and *lipL32*, which could lead to blockage of ribosome entry and thus to the inhibition of translation of these two major and abundant lipoproteins of the cell wall. However, these sRNAs were not detectable, probably because of their low abundance in the dRNA-seq data (Figure 4B). Nearly half of the sRNAs (7/13) were not detectable by Northern blot. Again, this may have been due to low abundance in the dRNA-seq data. The expression levels of these sRNAs were probably below the detection limit of our non-isotopic labeling method used.

*L. interrogans* contains Hfq-like (LMANv2_460028) and Rho (LMANv2_80086) homologs which in many bacteria stabilizes sRNA:mRNA base-pairing interactions. The genes encoding Hfq-dependent sRNAs usually possess a typical Rho-independent transcription terminator. However, the vast majority of the sRNAs detected in our study are not followed by a RIT and especially the number of asRNAs with RIT is marginal. Previous studies have shown that in some bacteria, the Hfq-dependent sRNAs may not contain RIT (Koo et al., 2011; Zeng and Sundin, 2014). Alternatively, Rho-dependent transcription termination may vary across the bacterial phylogeny and these termination sites can be difficult to predict using computational approaches in spirochetes.

In conclusion, we provide the first genome-wide TSS and promoter maps for the pathogen *L. interrogans*. Our approach defines TSS for most of the *L. interrogans* protein-coding genes and identifies sRNAs. Very little is known about sRNA and their potential regulatory actions in *Leptospira* spp. As more sRNAs become identified, efforts toward determining their functions will become imperative in the near future. The findings provided by this study will form the framework for future studies focused on defining the regulatory factors involved in promoting the adaptation of *L. interrogans* to the host, design of an artificial promoter system for gene studies, as well as the development of novel gene control technology, such as TALE and promoter control technology.

## Author contributions

Conceived and designed the experiments: MP, FY, CB, CM, AZ, and JC. Performed the experiments: AZ, PH, OS, JZ, AE, and CP. Contributed reagents/materials/analysis tools: MP, CB, and CM. Wrote the paper: MP. Revised the paper: AZ, LF, PH, CP, OS, AE, and FY.

### Conflict of interest statement

The authors declare that the research was conducted in the absence of any commercial or financial relationships that could be construed as a potential conflict of interest.

## Supplementary material

Supplementary Table 1 **Primers used for 5′ RACE experiments**.

Supplementary Table 2 **Probes used for validation of sRNA by Northern blot**. For putative sRNAs, a single biotinylated probe was designed complementary to the strand that contained the candidate sRNAs.

Supplementary Table 3 **RNA-seq coverage of ***L. interrogans*** genes 30 and 37°C**.

Supplementary Table 4 **Transcriptional Start Sites (TSS) and operons in ***L. interrogans*****.

Supplementary Table 5 **Shine-Dalgarno-like sequences identified near initiation codons of genes with pTSS from ***L. interrogans*****.

Supplementary Table 6 **Search of conserved motifs in the 80 to +1 sequences upstream of the detected pTSS at 30 and 37°C**.

Supplementary Table 7 **Search of RpoD, RpoE, and RpoF binding sites in the 80 to +1 sequences upstream of the detected pTSS at 30 and 37°C**.

Supplementary Table 8 **List of predicted RpoN (σ24) binding sites at 30 and 37°C**.

Supplementary Table 9 **List of small RNA detected in ***L. interrogans*** at 30 and 37°C**.

Supplementary Table 10 **Prediction of minimal free energy of sRNA secondary structure**.
