# Supplementary material for: Genome-Wide Transcriptional Start Site Mapping and sRNA Identification in the Pathogen Leptospira interrogans
Source: Front Cell Infect Microbiol. 2017 Jan 19;7:10. doi: 10.3389/fcimb.2017.00010 (PMC5243855; doi:10.3389/fcimb.2017.00010)
Supplement: Supplementary Table 1 — Primers used for 5′ RACE experiments. [file Table1.DOCX]

**Supplementary Table 1**: Primers used for 5’RACE experiments.

| **primer** | **gene** | **sequence (5’- 3’)** |
| --- | --- | --- |
| hfq GSP1 | LMANv2_460028 | TTGAGCTGCGTCTTT |
| hfq GSP2 | LMANv2_460028 | AGCTTGATAATCTTGGCGGGAATGA |
| LipL32 GSP1 | LMANv2_150111 | AATGTGGCATTGATT |
| LipL32 GSP2 | LMANv2_150111 | GCTTTGAAAGCGTCGCTTACTAAGT |
| mreB GSP1 | LMANv2_160018 | GAACAGCACGTCTTT |
| mreB GSP2 | LMANv2_160018 | GAACTCCGATCACAATTCTAGGTTT |
| hemO GSP1 | LMANv2_680004 | ACGCGGTACGATAATT |
| hemO GSP2 | LMANv2_680004 | CCATTGATGTCTTGAATCATAGGAA |
| PerR GSP1 | LMANv2_280031 | CCTAAATTCTGGCTTTT |
| PerR GSP2 | LMANv2_280031 | AAGACCCAATTCTAAATTCAAGGATT |
| 110011GSP1 | LMANv2_110011 | AGTTCTTCCAGGAATATT |
| 110011GSP2 | LMANv2_110011 | GGTGGAATGTTCTGCAATTGATTTA |
| 1850003GSP1 | LMANv2_60079 | CTCTGTAGTTCTGATTGATT |
| 1850003GSP2 | LMANv2_60079 | GCCATCATCATATATTGCATTTGAGAATT |
| 150128GSP1 | LMANv2_150128 | TTAACTACGGCCAGAATA |
| 150128GSP2 | LMANv2_150128 | GCTTTCACGGATGATTAGATATTCTT |
| 370081GSP1 | LMANv2_370081 | GTAGGAAACGTATCGTTT |
| 370081GSP2 | LMANv2_370081 | TTGAGCCTTGTTAACATCGTCATTT |
| 580002GSP1 | LMANv2_580002 | TAATCCTTAGATTTTTCTG |
| 580002GSP2 | LMANv2_580002 | TGCTTTCATCGTTTTCTTTCCTTCGT |
