# Supplementary material for: Genome-Wide Transcriptional Start Site Mapping and sRNA Identification in the Pathogen Leptospira interrogans
Source: Front Cell Infect Microbiol. 2017 Jan 19;7:10. doi: 10.3389/fcimb.2017.00010 (PMC5243855; doi:10.3389/fcimb.2017.00010)
Supplement: Supplementary Table 2 — Probes used for validation of sRNA by Northern blot. For putative sRNAs, a single biotinylated probe was designed complementary to the strand that contained the candidate sRNAs. [file Table2.DOCX]

**Supplementary Table 2**: Probes used for validation of sRNA by Northern blot

For putative sRNAs, a single biotinylated probe was designed complementary to the strand that contained the candidate sRNAs.

| **Name** | **Probe sequence (5’- 3’) *** | **Expected size (nt)** |
| --- | --- | --- |
| sRNA_30_26 | GGTTAATTTTGTTTGTGATTTCCAC | 139 |
| sRNA_30_275 | AATCTCTACAGCGTAAAGCAATGTC | 76 |
| sRNA_30_373 | GGCTCATAACCCAAAGGTCATA | 51 |
| sRNA_30_292 | GTAGTCTCCTTTCGATTGTGTTTGT | 115 |
| sRNA_30_254 | CTTTCAGGAATTTTCATAGGTCGTA | 173 |
| sRNA_30_180 | TCACTTTTATTCGGTTTTCTTTTTG | 188 |
| sRNA_30_204 | GTTCTGCGATTAGAAACAACA | 208 |
| sRNA_30_255 | TACGTCCTTTTGGAAGTTTGACTAC | 212 |
| sRNA_30_271 | ACATCCTTCATCTTTCTTTTCAGTG | 104 |
| sRNA_30_192 | CGTTCTGTTGAGTTCATTTTGATTC | 90 |
| sRNA_30_225 | TCAAGGAGAAAATAATGATCAATAGAC | 51 |
| sRNA_37_177 | AAAAACTTTCGATTTTGGCTATCTC | 51 |
| sRNA_30_33 | TTTTTCTCGGACTTAGTTTCTTTTG | 304 |
| RNase P | GTATCTTTTCTGTTGCACTTTCCATATT | 427 |
| sRNA_30_4 | CTCTATTACTAGATGAGTTGATACAC | 102 |

* 5´ Biotinylated
